# Impact of Zone 2 preemptive TEVAR on treatment strategy in uncomplicated type B aortic dissection: a 10-year outcome, study of 29 cases with Zone 2 placement

**DOI:** 10.1007/s11748-026-02276-w

**Published:** 2026-03-27

**Authors:** Ken Nakamura, Kentaro Akabane, Kimihiro Kobayashi, Shusuke Arai, Miku Konaka, Jun Hayashi, Cholsu Kim, Hideaki Uchino, Takao Shimanuki, Tetsuro Uchida

**Affiliations:** 1 Division of Cardiovascular Surgery, Nihonkai General Hospital, 30 Akihochou, Sakata City, Yamagata, Japan; 2 Second Department of Surgery, Faculty of Medicine, Yamagata University, Yamagata, Japan

**Keywords:** Uncomplicated stanford type B aortic dissection, Best medical treatment, Preemptive thoracic endovascular aortic repair, Aortic aneurysm, Aortic remodeling

## Abstract

**Objective:**

The indication for preemptive thoracic endovascular aortic repair (TEVAR) is expanding due to improved outcomes in uncomplicated type B aortic dissection (TBAD) compared with best medical therapy (BMT). Long-term outcomes and future treatment strategies for TBAD managed with preemptive TEVAR will be reported.

**Subjects and methods:**

Of 432 patients with acute TBAD treated at two centers between July 2004 and August 2024, 324 patients who completed acute BMT (1–237 months) were included. Their outcomes were compared with those of patients who did not undergo TEVAR.

**Results:**

Of 324 patients, 277 (control group: CTR) continued BMT and 47 (Zone 2 landing n = 29, Zone 3 landing n = 7, others n = 11) underwent preemptive TEVAR. Propensity score matching was performed to compare the Zone 2 TEVAR group (Z2) with the CTR. There were more patients with patent false lumen in the Z2 (41% vs. 83%, *p* < *0.05*). Pre- and post-TEVAR aortic diameters had decreased from 43.8 ± 6.8 mm to 40.2 ± 10.0 mm (*p* < *0.001*) at 12 months, the same value as the CTR (40.2 ± 11.7 mm). The aortic event free rate (CTR vs. Z2, 1/5/10 years) was 76/69/69% vs. 100/96/96%, respectively, (*p* < *0.05*). Although two patients (7.1%) had aortic events (enlargement of aneurysms) after TEVAR, no aorta-related deaths.

**Conclusion:**

Among patients who met the anatomic criteria, long-term outcomes of Zone 2 TEVAR performed during the subacute and early chronic phases were favorable. Preemptive TEVAR may be considered a viable treatment strategy for managing the aortic arch in the early stages of TBAD.

## Introduction

Acute aortic dissection, the most common catastrophic aortic event, occurring at a frequency of 3.6–10% per 100,000 population in Japan [1, 2]. In 2021, 2693 patients with aortic dissection underwent thoracic endovascular aortic repair (TEVAR), with a hospital mortality rate of 1.9% for chronic cases, which is improving over time [2]. With improvements in TEVAR outcomes for aortic dissection and the emergence of new evidence, preemptive TEVAR has recently attracted increasing attention [1, 3]. Ten years have passed since the European Society of Cardiology guidelines upgraded preemptive TEVAR to class IIa recommendation [3]. Since then, it has been increasingly recognized as an effective treatment option for uncomplicated type B aortic dissection (TBAD), offering improved prognosis compared to conservative management, and its indications are continuing to expand [4, 5]. In the United States, Thoracic Branch Endoprosthesis has allowed for safer and more efficient deployment of the aortic arch, however TEVAR in the arch is known to have increased risks [6], and further development in this area is warranted. We have been performing preemptive TEVAR, including procedures with Zone 2 landing, for over a decade and herein report our outcomes as a proposed treatment strategy for managing aortic arch involvement in TBAD.

## Subjects and methods

The institutional ethical review board approved the research protocol and patient written consent were waived because of the retrospective nature of the study (Institutional Review Board #2018–245). All patients were informed about the use of their data for clinical research.

**Fig. 1:**
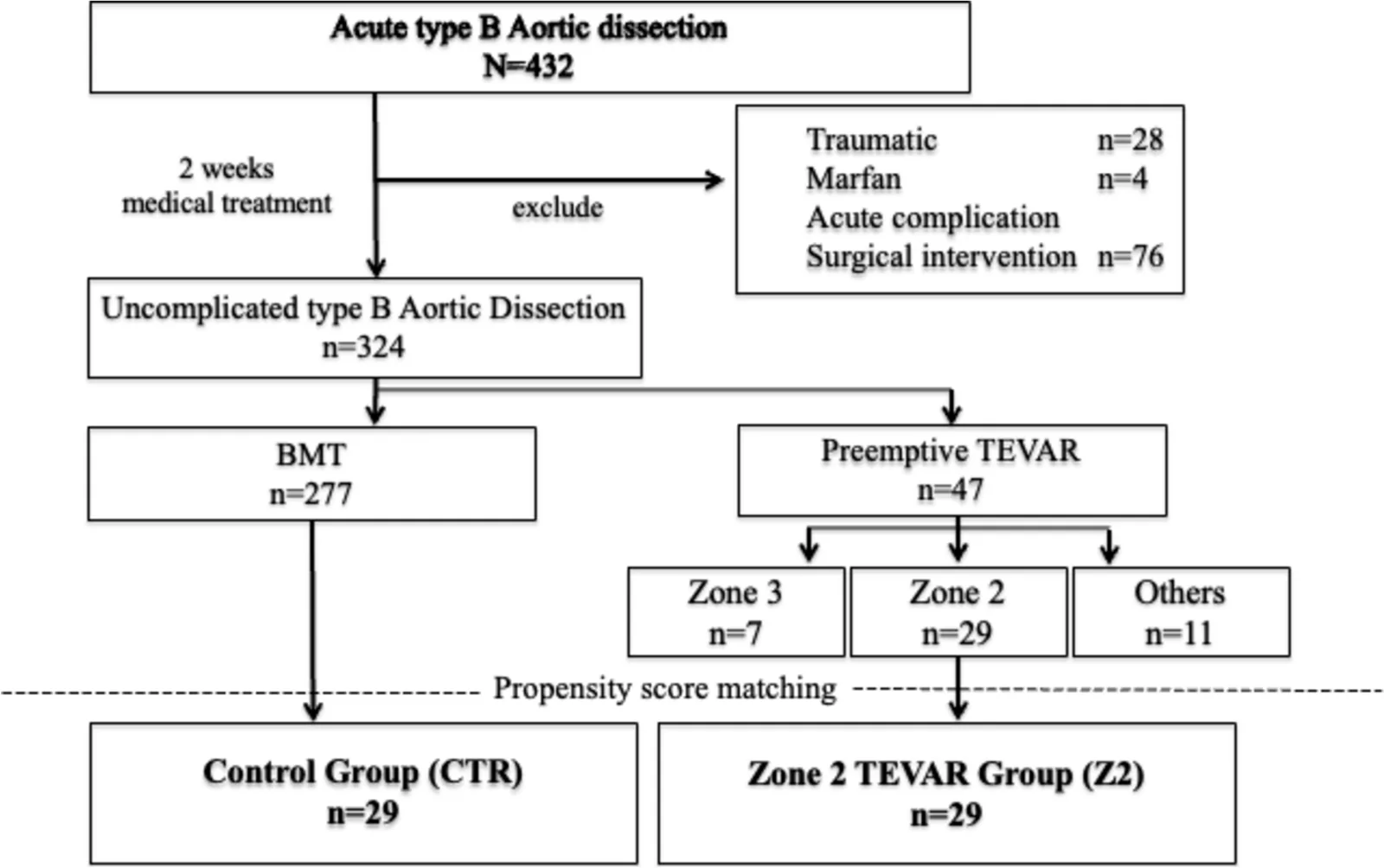
Flow diagram of the entire series of patients with Stanford type B aortic dissection. The study comprised 432 consecutive patients with acute type B aortic dissection. Patients with uncomplicated type B aortic dissection (n = 324) were divided into best medical therapy (BMT) group and preemptive TEVAR group. Propensity score matching was performed and adjusted for the background characteristics of each patient group. Patients were divided into 2 groups according to the treatment option, control group (CTR) consisted of 29 patients who received BMT, and Zone 2 TEVAR Group (Z2) consisted of 27 patients who underwent preemptive TEVAR

Two centers in Yamagata prefecture participated in this study. Data were collected between July 2004 and August 2024 in Yamagata University Hospital and between February 2016 and January 2025 in Nihonkai General Hospital (Fig. 1). Acute aortic dissection was defined as a condition in which the patient presented with pain and other symptoms as the primary complaints. Acute uncomplicated TBAD was defined as the absence of malperfusion (both dynamic obstruction, which improves with false lumen decompression, and static obstruction, which does not improved), as well as the absence of signs of early disease progression, such as type A dissection occurring within 14 days of symptom onset [1]. As outlined in the guidelines, the course of aortic dissection is classified into acute (< 14 days), subacute (15–90 days), and chronic (> 90 days) phases [1, 3]. Upon admission, patients were managed in either the intensive care unit (ICU) or a high care unit (HCU). All patients underwent contrast-enhanced computed tomography (CT) scanning at emergency admission, and on the 1st and 7th days following admission. Patients were eligible for discharge 4 weeks after onset. This protocol was developed based on the Japanese guidelines [1]. During follow-up, patients who experienced an aorta-related event, despite medical management, underwent an aortic intervention. The term “aorta-related event” refers to any of the following: enlargement of the aortic diameter (≥ 55 mm or ≥ 5 mm enlargement within 6 months), malperfusion, or aortic rupture [1, 3]. CT angiography (CTA) images obtained at presentation, as well as follow-up CT scans, were reviewed for all patients. The standard imaging protocol included scans at symptom onset, at discharge, 3 or 6 months post-discharge, 12 months post-discharge, and annually thereafter. The scan regimen varied for individual patients based on their findings. All imaging studies were reviewed for radiological signs of adverse events, such as organ malperfusion or rupture, as well as for evidence of pathological resolution. These measures follow the same methodology as described in our previous report [5, 7, 8]. Preemptive TEVAR was defined as TEVAR prior to the occurrence of an aortic adverse event, as defined in the guideline and in previous reports [1, 3, 5]. The indication for preemptive TEVAR was determined comprehensively based on individual patient risk factors, including an aortic diameter ≥ 40 mm, a narrow true lumen, a large or enlarging false lumen, a patent false lumen, and a large primary entry (≥ 10 mm), together with the patient’s informed and proactive request after sufficient explanation. Stent grafts were placed with a minimum of 2 cm of non-dissected, healthy aorta proximally. In Zone 2 TEVAR, debranching was selectively performed prior to stent placement when complete coverage of the left subclavian artery (LSA) and subsequent loss of antegrade LSA flow were anticipated; revascularization was not performed when only partial LSA coverage was expected and antegrade flow could be preserved. The devices were selected to match the diameter of the proximal aorta and were all deployed in the true lumen. A total of 324 CT images, along with their analysis were available for routine surveillance. Image analysis was performed on a SYNAPSE VINCENT system (Tokyo Japan, FUJIFILM Holding Corporation) with a dedicated 3D image analyzer. All CTA measurements were obtained using multiplanar reconstruction (MPR) images in an axial plane perpendicular to the aortic centerline. The aortic centerline was generated using software on the VINCENT system, which employs a 3D algorithm to perform MPR centered on the contrast-enhanced aortic lumen. In no case did the maximum aortic diameter occur in a non-dissected segment. The diameters of both the true and false lumen were measured. Measurements were performed on the orthogonal cross-section where the aortic diameter was maximal. Aortic growth rate was calculated by dividing the change in aortic diameter by the time interval (six months or one year) from symptom onset. The status of the false lumen on imaging was classified as “patent” if there was flow without thrombus, and as “partial thrombosis” in the later phase of contrast-enhanced CT.

The clinical characteristics and CT imaging parameters of patients at emergency admission and during follow-up were summarized. Categorical variables are presented as percentages, while continuous variables are expressed as medians with interquartile ranges. Outcomes were assessed with unadjusted and multivariable models. Aorta-related event-free rates were calculated using Kaplan– Meier curves and examined by the log-rank test. All model-based results are presented with 95% confidence intervals. This study was performed under intention-to-treat analysis. A matched group analysis was performed by propensity matching preemptive TEVAR cases and control (CTR) cases. Propensity scores were generated using logistic regression analyses in 2 steps. Potential predictors were selected from a published data review, known confounding covariates for the outcomes of interest, differences between the 2 patients groups (Table 1), and clinical judgement. The groups were matched with age, sex, body mass index, aortic diameter in early subacute phase, history of chronic obstructive pulmonary disease, hypertension, diabetes mellitus and hyperlipidemia. Univariate analysis was performed using ANOVA or Student’s t-test and the Chi-square test. The analysis of aortic diameter with follow-up outcomes was calculated with Repeated Measures Analysis of Variance (RMANOVA). All statistical analyses were performed with JMP software, ver. 18 (SAS Institute, Tokyo, Japan).

**Table 1:** Baseline patient characteristics of all patients with or without Zone 2 preemptive TEVAR

Characteristics	All patients (n = 324)	After Propensity Score Matching		
		CTR (n = 29)	Zone 2 Preemptive TEVAR (n = 29)	*P* Value
Age, y, Mean ± SD	70.1 ± 12.1	68.3 ± 11.4	65.7 ± 10.1	0.37
Age < 60, %	21 (67 of 324)	24 (7 of 29)	21 (6 of 29)	1.00
Male, %	71 (230 of 324)	76 (22 of 29)	76 (22 of 29)	1.00
Height, cm, Mean ± SD	161.2 ± 9.5	161.1 ± 9.3	163.8 ± 9.5	0.26
Weight, kg, Mean ± SD	61.9 ± 14.4	67.6 ± 14.4	70.4 ± 16.7	0.51
BMI (kg/m^2^), Mean ± SD	23.7 ± 4.3	25.9 ± 4.2	26.1 ± 5.0	0.86
COPD, %	40 (128 of 324)	31 (9 of 29)	27 (8 of 29)	1.00
HTN, %	98 (317 of 324)	100 (29 of 29)	100 (29 of 29)	–
Diabetes Mellitus, %	8.0 (26 of 324)	24.1 (7 of 29)	13.8 (4 of 29)	0.51
History of stroke, %	7.1 (23 of 324)	6.9 (2 of 29)	3.5 (1 of 29)	1.00
Renal insufficiency, %	13.0 (42 of 324)	24.1 (7 of 29)	10.3 (3 of 29)	0.29
Coronary artery disease, %	4.3 (14 of 324)	0 (0 of 29)	6.9 (2 of 29)	0.49
Hyperlipidemia, %	32 (103 of 324)	52 (15 of 29)	45 (13 of 29)	0.79
Follow up period, months, Mean ± SD	75 ± 54	81 ± 59	76 ± 43	0.72
*Acute course of disease*				
Delirium, %	46 (145 of 317)	25.9 (7 of 29)	51.7 (15 of 29)	0.06
Delirium onset, day %	2.4 (1.0–3.0)	2.8 (1.2–4.4)	2.4 (1.1–3.7)	0.68
NPPV required, %	26 (82 of 317)	33.3 (9 of 29)	41.4 (12 of 29)	0.59
Tracheal Intubation, %	8 (25 of 317)	11.1 (3 of 27)	10.3 (3 of 29)	1.00
Positive pressure ventilation required (NPPV or mechanical ventilation), %	29 (91 of 316)	44.4 (12 of 27)	42.9 (12 of 28)	1.00
ICU + HCU stay, day Mean ± SD	6.2 ± 4.5	8.0 ± 6.8	6.2 ± 3.7	0.29
*Medication at discharge from the hospital*				
Beta blockers n,%	80 (245 of 307)	88 (23 of 26)	77 (20 of 26)	0.47
Angiotensin converting enzyme inhibitors,%	18 (54 of 307)	12 (3 of 26)	15 (4 of 26)	1.00
Angiotensin II receptor blockers,%	61 (188 of 306)	65 (17 of 26)	65 (17 of 26)	1.00
Calcium channel blockers,%	79 (243 of 307)	88 (23 of 26)	73 (19 of 26)	0.29
alpha blockers, %	29 (89 of 307)	27 (7 of 26)	42 (11 of 26)	0.38
Thiazide	4 (13 of 307)	8 (2 of 26)	4 (1 of 26)	1
loop diuretics, %	5 (15 of 307)	12 (3 of 26)	5 (15 of 307)	1
aldosterone antagonist, %	7 (22 of 307)	15 (4 of 26)	0 (0 of 26)	0.11
statins,%	38 (117 of 307)	62 (16 of 26)	35 (9 of 26)	0.10
Steroids,%	4 (11 of 311)	4 (1 of 26)	4 (1 of 26)	1.00
Anticoagulants,%	11 (35 of 312)	15 (4 of 26)	4 (1 of 26)	0.35

TEVAR, thoracic endovascular aortic repair; CTR, control; SD, standard deviation; BMI, body mass index; COPD, chronic obstructive pulmonary disease; HTN, hypertension; NPPV, noninvasive positive pressure ventilation; ICU, intensive care unit; HCU, high care unit

## Results

**Fig. 2:**
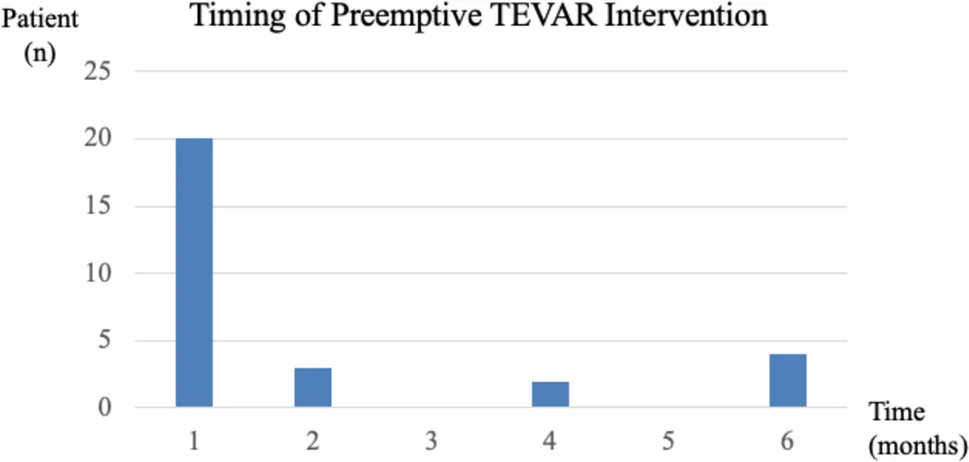
Timing of intervention in the Zone 2 preemptive TEVAR group (n = 29). Twenty patients (69%) underwent TEVAR within 1 month after onset, and most procedures were performed during the subacute phase

**Fig. 3:**
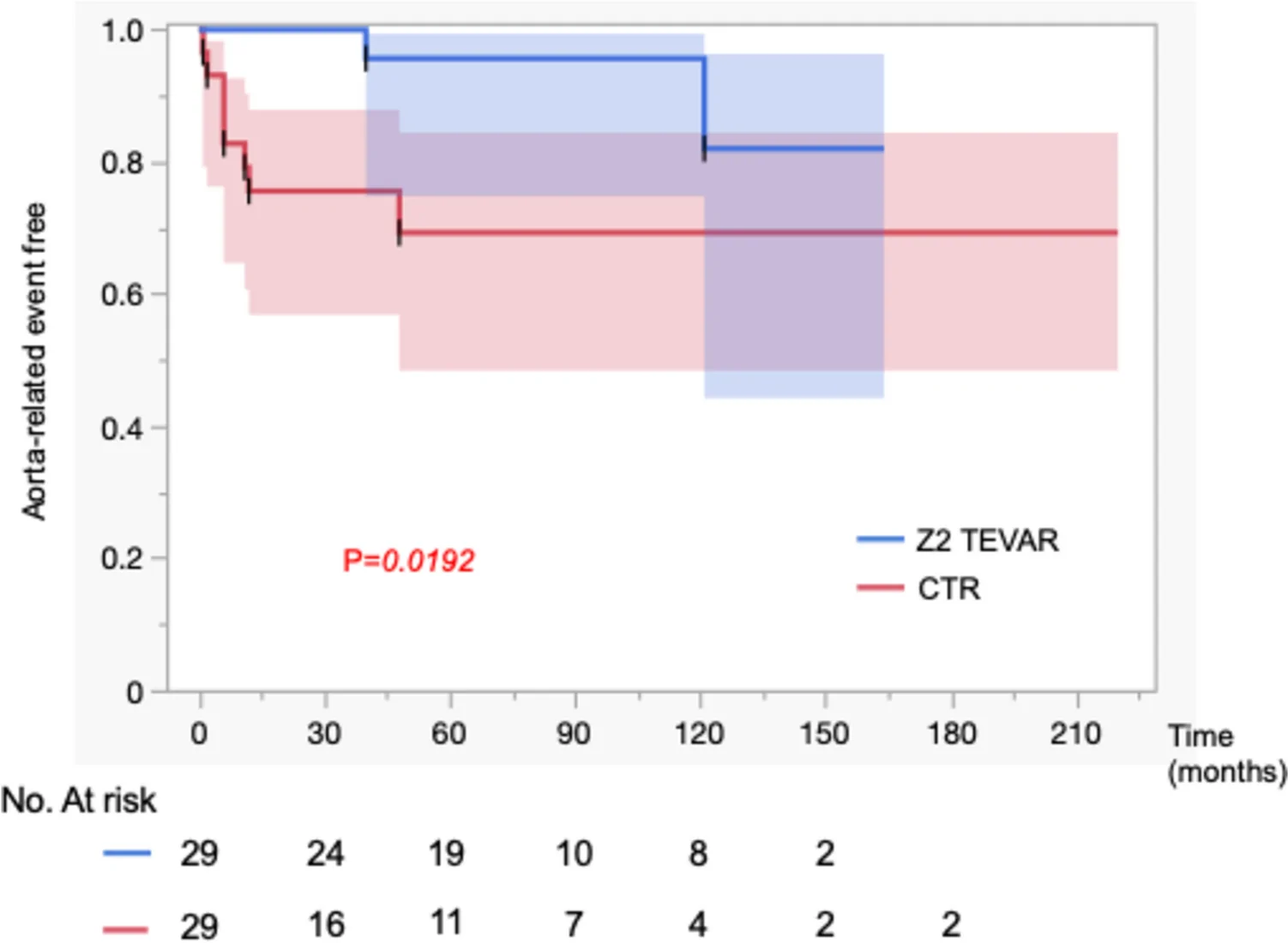
Kaplan–Meier curve of freedom from aorta-related event for 58 patients. Propensity score matching was performed and adjusted for the background characteristics. Zone 2 TEVAR was performed in 29 patients (Z2). In the control group (CTR, n = 29), Best medical therapy was performed on the basis of conservative treatment, and if aortic adverse events occurred during the course of the disease, graft replacement was performed

**Fig. 4:**
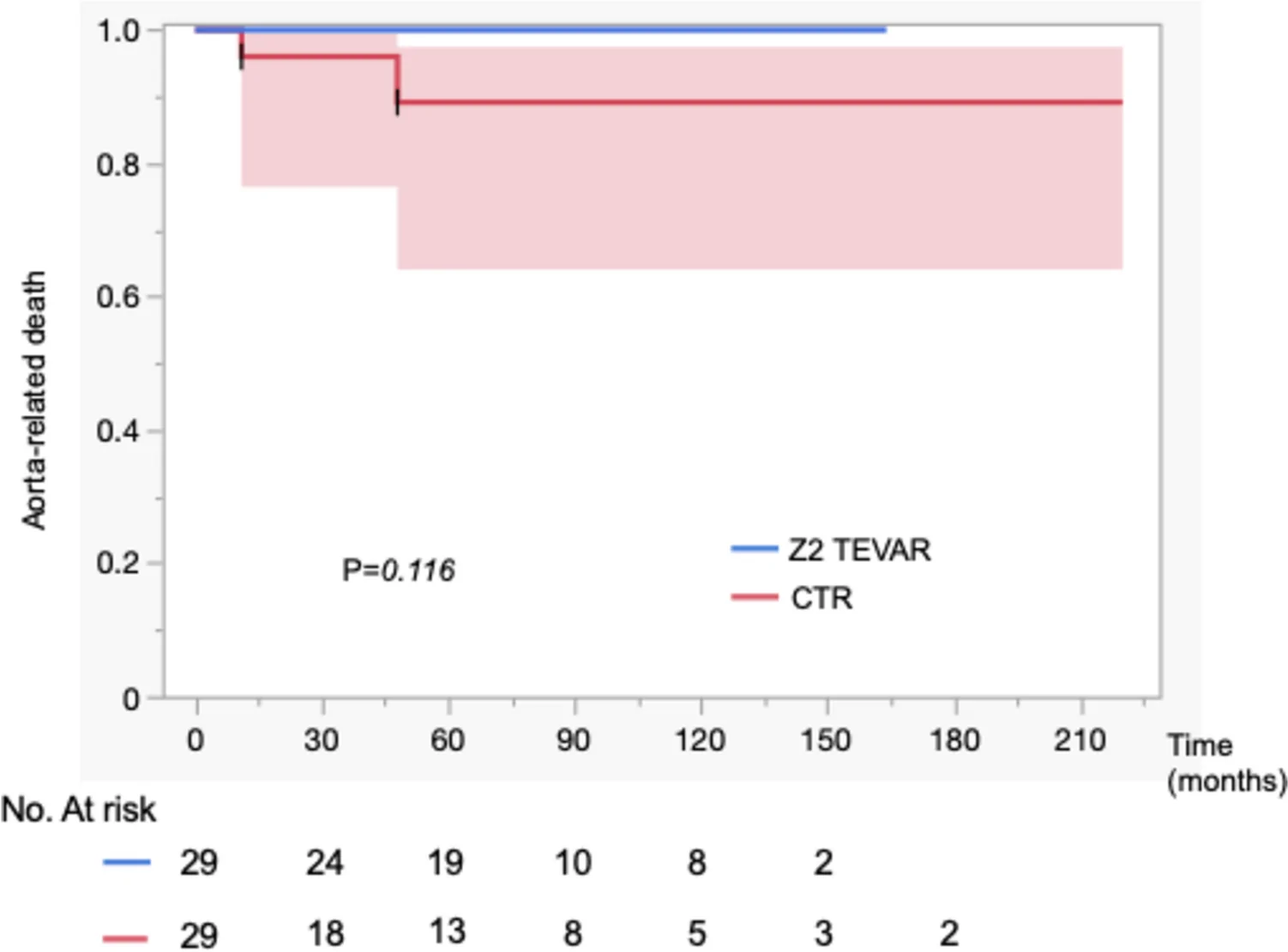
Kaplan–Meier curve of freedom from aorta-related death for 58 patients. Propensity score matching was performed and adjusted for the background characteristics. The Zone 2 preemptive TEVAR group (Z2, n = 29) was compared to a control group (CTR, n = 29) that continued best medical therapy

**Fig. 5:**
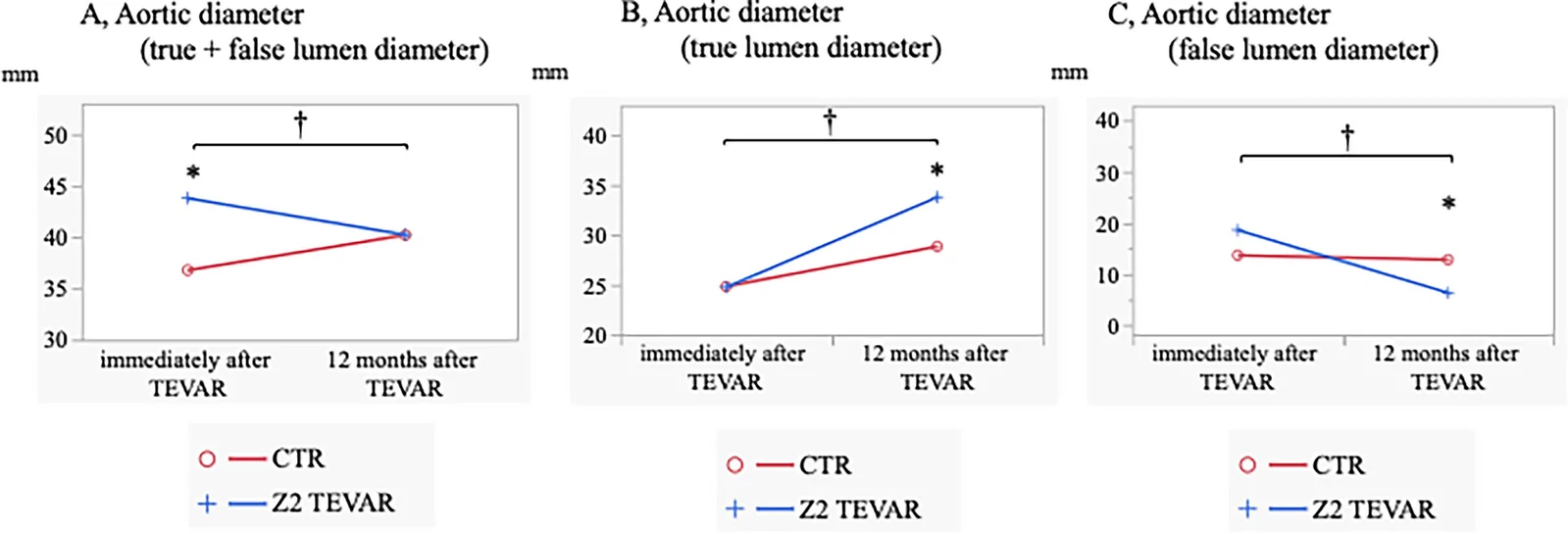
Aortic diameter before intervention and 12 months after Zone 2 Preemptive TEVAR (Z2 TEVAR, n = 29). Compared to the control (CTR) group (n = 29) after matching. **A** Change in aortic diameter (true and false lumen diameters), *represents significant difference between CTR and Z2 TEVAR (< 0.0001). †Represents significant difference between before preemptive TEVAR and 12 months after TEVAR (< 0.001). **B** Change in aortic diameter (true lumen diameters), *represents significant difference between CTR and Z2 TEVAR (< 0.0001). †Represents significant difference between before preemptive TEVAR and 12 months after TEVAR (< 0.0001). **C** Change in aortic diameter (false lumen diameters), *represents significant difference between CTR and Z2 TEVAR (< 0.0001). †Represents significant difference between before preemptive TEVAR and 12 months after TEVAR (< 0.001)

A total of 432 patients were admitted with a diagnosis of acute TBAD. Among them, 324 patients were enrolled in the study, all of whom had been treated with medical therapy during the acute phase and continued on best medical therapy (BMT) during the subacute phase. Twenty patients did not undergo follow-up CT after the subacute phase, 10 of whom underwent plain CT. All were Japanese, and 230 (71%) were males. The mean age of all patients was 70.1 (34–96) years. The most common comorbidity was hypertension (n = 317, 98%); diabetes mellitus and renal insufficiency were present in 8.0% (26 of 324) and 13.0% (42 of 324) of the study population, respectively. The mean follow-up was 75 ± 54 months (Table 1). Surgical intervention was required due to aortic-adverse events during follow-up in 67 patients (20.7%), and preemptive TEVAR was performed in 47 (Zone 2 29 cases, Zone 3 7 cases, others 11 cases) of them (14.5%). All patients who underwent preemptive TEVAR showed aortic enlargement; however, none met the criterion of a maximum aortic diameter > 55 mm, as the enlargement occurred over a short period. There were no cases in which stent grafts were placed in the aortic arch proximal to Zone 2 as part of preemptive TEVAR. Eleven patients (3.4%) died due to aortic adverse events, whereas no aorta-related deaths occurred among the 47 patients who underwent preemptive TEVAR. The Zone 2 TEVAR group (Z2: n = 29) had a mean age of 65.7 (61.7–69.7) years, mean BMI of 26 ± 5, 22 men (76%), and mean follow-up of 76 ± 43 months. Demographics, comorbid conditions, and dissection characteristics based on the initial CT findings were compared between groups with propensity score matching (Tables 1, 2). There were no significant differences in patient background characteristics. In the Z2, the false lumen patency was 83% (*p* < 0.05), the mean diameter of the intimal tear was 11.7 ± 5.9 mm, and the average number of tears was 2.7 ± 1.6 (Table 2). TEVAR was performed in the subacute phase in 79% of cases and in the early chronic phase in 21%; notably, 69% (20 cases) underwent early intervention within 1 month after onset (Fig. 2). Complete thrombosis of the false lumen was achieved in all patients, with a mean time to thrombosis of 2.5 ± 4.7 months (Table 2). Perfused with partial thrombosis was significantly more common in the Z2 (14% [4 of 29] vs. 31% [9 of 29], respectively, *p* < 0.01) and aortic growth rate was also significantly higher (0.7 ± 1.2 vs 1.9 ± 1.8, respectively, *p* < 0.01) (Table 2). In addition, other well-established risk factors for late aortic events, such as initial aortic diameter ≥ 40 mm in the acute phase, entry tear located on the lesser curvature, and age < 60 years, were evaluated; however, no significant differences were observed between the two groups. The mean extent of stent graft coverage was 188 ± 47 mm, and few cases required coverage exceeding 200 mm. Cook devices were the most frequently used, accounting for 62% (18 of 29) of all cases (Table 3). The Kaplan–Meier curve of freedom from aorta-related death after matching showed no significant difference between two groups, and there were no deaths after the Z2 TEVAR (n = 29). (Fig. 3). Cases requiring an adequate proximal landing zone were considered indications for debranching, and axillary artery bypass was performed prior to TEVAR. These procedures were carried out in 13 patients (45%, 13 of 29), with no complications such as cerebral infarction. The aorta-related event free (CTR vs. Z2, 1/5/10 years) was 76/69/69% vs 100/96/96% (p < 0.05) (Fig. 4), respectively, and 2 patients (7.1%) had aorta-related events (aneurysms) after preemptive TEVAR. One patient had type Ia endoleak due to migration 10 years after TEVAR, and the other had an enlarged aortic arch aneurysm due to blow-up 40 months after TEVAR, both patients underwent total arch replacement. In the control group, 11 patients experienced aorta-related adverse events, all of whom showed aneurysmal dilatation of ≥ 5 mm within 6 months or a maximum diameter ≥ 55 mm. Aorta-related deaths occurred in two patients who refused surgical intervention and died of rupture, while four patients underwent TEVAR and three underwent open graft replacement, all of whom survived. Thus, compared with the preemptive TEVAR group, the control group had a higher incidence of aortic events and a greater number of patients requiring invasive treatment. The aortic diameter before and after TEVAR decreased from 43.8 ± 6.8 mm to 40.2 ± 10.0 mm at 12 months (p < 0.001), which was similar to the CTR (40.2 ± 11.7 mm) (Fig. 5A). Before TEVAR, the false lumen aortic diameter was significantly larger in the Z2 (CTR vs. Z2; 12 ± 7 vs. 17 ± 7, respectively, p = 0.048), and after TEVAR, the true lumen aortic diameter was increased (immediately after TEVAR vs. 12 months after TEVAR; 24.8 ± 10.3 vs. 33.8 ± 4.7, respectively, p < 0.0001) and the false lumen aortic diameter was decreased (immediately after TEVAR vs. 12 months after TEVAR; 18.7 ± 8.8 vs. 6.4 ± 9.1, respectively, p < 0.0001), each with a significant difference compared to the CTR (*p* < 0.001) (Table 4, Fig. 5B, C).

**Table 2:** Initial computed tomography findings for all patients with or without Zone 2 preemptive TEVAR

Characteristics	All patients (n = 324)	After Propensity Score Matching		
		CTR (n = 29)	Zone 2 Preemptive TEVAR (n = 29)	*P* Value
Aortic diameter in early subacute, mm, Mean ± SD	38 ± 6	39 ± 7	39 ± 5	0.95
Initial aortic diameter ≥ 40 mm in the acute phase, %	33 (108 of 324)	38 (11 of 29)	55 (16 of 29)	0.29
True lumen aortic diameter (mm)	25 ± 7	26 ± 9	22 ± 8	0.11
False lumen aortic diameter (mm)	12 ± 6	12 ± 7	17 ± 7	0.05
Rate of true/false lumen diameter (TLD/FLD)	3.2 ± 3.8	3.8 ± 4.5	1.8 ± 1.6	0.03
Entry located distal arch, %	80 (248 of 311)	85 (23 of 27)	90 (26 of 29)	0.70
Entry located tracheal bifurcation level, %	5 (16 of 311)	11 (3 of 27)	7 (2 of 29)	0.66
Entry located diaphragm level, %	9 (28 of 311)	7 (2 of 27)	3 (1 of 29)	0.60
Entry located celiac artery level, %	4 (12 of 311)	4 (1 of 27)	0 (0 of 29)	0.48
Entry located abdominal aorta, %	3 (9 of 311)	0 (0 of 27)	0 (0 of 29)	-
Number of intimal tears	2.0 ± 1.3	2.4 ± 1.3	2.7 ± 1.6	0.42
Diameter of primary entry, mm, Mean ± SD	10.8 ± 6.6	11.0 ± 5.6	11.7 ± 5.9	0.73
Patent false lumen, %	41 (133 of 324)	41 (12 of 29)	83 (24 of 29)	0.003
Perfused with partial thrombosis, %	19 (60 of 324)	14 (4 of 29)	31 (9 of 29)	0.005
Entry tear on the lesser curvature, %	22 (71 of 324)	17 (5 of 29)	24 (7 of 29)	0.75
*Follow-up findings*				
Aortic growth rate (mm/ month)	0.50 ± 1.33	0.7 ± 1.2	1.9 ± 1.8	0.003

CTR, control; TEVAR, thoracic endovascular aortic repair; SD, standard deviation

**Table 3:** Findings after Zone 2 preemptive TEVAR

Valuables	Zone 2 Preemptive TEVAR (n = 29)
PETTICOAT technique, %	26 (6 of 29)
Stent graft coverage, mm	188 ± 47
*Device*	
Cook, %	62 (18 of 29)
Medtronic, %	3 (1 of 29)
GORE, %	21 (6 of 29)
Terumo, %	14 (4 of 29)

TEVAR, thoracic endovascular aortic repair

**Table 4:** Changes in aortic diameter. Subanalysis of post zone 2 preemptive TEVAR

		Aortic diameter, mm, Mean ± SD			
		Early subacute	Just before preemptive TEVAR	Immediately after Zone 2 TEVAR	12 months after Zone 2 TEVAR
CTR	True + False lumen	39.4 ± 6.9		36.8 ± 8.8	40.2 ± 11.7
	True lumen	25.7 ± 9.4		24.8 ± 9.5	28.9 ± 8.8
	False lumen	12.0 ± 6.8		13.7 ± 8.7	12.9 ± 11.0
Zone 2 TEVAR	True + False lumen	39.3 ± 4.9	43.9 ± 6.7	43.8 ± 6.8	40.2 ± 10.0
	True lumen	21.9 ± 8.1	22.7 ± 10.7	24.8 ± 10.3	33.8 ± 4.7
	False lumen	17.5 ± 6.9	20.7 ± 9.1	18.7 ± 8.8	6.4 ± 9.1

TEVAR, thoracic endovascular aortic repair; CTR, control; SD, standard deviation

## Discussion

Subacute TBAD is known to be more prone to aortic adverse events in patients with refractory hypertension, large aortic diameter (≥ 40 mm), the number of vessels originating from the false lumen and the number of intercostal arteries, entry tear on the lesser curvature, and a false lumen diameter > 22 mm [6, 8–10]. Symptomatic cases (e.g., refractory hypertension, recurrent or persistent pain) are indicated for urgent treatment, while asymptomatic, uncomplicated, high-risk cases have the option of preemptive TEVAR [1, 3]. Since there is no significant risk in continuing conservative treatment instead of undergoing TEVAR, preemptive TEVAR should be performed more carefully to prevent complications. The most important considerations to prevent serious complications such as retrograde type A dissection (RTAD) and paraplegia are (I) adequate informed consent, (II) careful consideration of the indications for the procedure in the aortic arch, (III) avoid oversizing, (IV) avoid touch-ups, and (V) keep a healthy landing on the proximal side, and so on [7]. In the INSTEAD trial, the timing of intervention within one year of the onset of TBAD resulted in a better prognosis in the TEVAR group [11]. We have mainly performed TEVAR in the subacute phase, at the latest within 6 months. Recently, there have been reports that subacute TEVAR had a better prognosis than acute TEVAR [12]. Even with the above precautions, it is known that RTAD is four times more likely to occur in Zone 2 than in Zone 3 (2.66% vs. 0.67%, respectively), so careful application is required when performing preemptive TEVAR [6, 13]. Because preemptive TEVAR is a procedure in which complications must be strictly avoided, we considered it inappropriate to analyze cases across different landing zones with inherently distinct risk profiles. Zone 2 TEVAR, in particular, presents unique technical and anatomical challenges, including the need for adequate proximal sealing in the aortic arch, the risk of endoleak, and potential cerebrovascular complications due to the involvement of supra-aortic branches. Therefore, to better characterize the essential risks and optimal treatment strategies of preemptive TEVAR, our study specifically focused on Zone 2 cases. We did not experience any acute complications in this study, which may be related to the fact that the intervention was performed before the aortic diameter had expanded beyond 55 mm [7]. This is presumably because the proximal site of the stent graft is close to the normal aortic diameter and the structure is preserved. Therefore, even with preemptive TEVAR, we believe that debranching and axillary artery bypass, if necessary, are effective for safe procedures in order to achieve a sufficiently healthy landing. In our series, a healthy proximal landing zone of at least 2 cm was secured in all cases; however, because the distance to the left subclavian artery (LSA) orifice was often less than 2 cm, antegrade LSA flow was generally preserved, and no major cerebrovascular complications were observed. This anatomical relationship explains why some Zone 2 cases did not require revascularization, even though sufficient proximal fixation was achieved. Recent reports from Japan indicate that aorta-related death and aortic adverse events occurred in nearly 30–35% of medically managed TBAD patients within 5 years [8, 14], which compares favorably with other countries [3]. Although the INSTEAD XL is the most well-known report on the mid-term outcome of preemptive TEVAR [11], there are many impressive results reported to date. Meta-analysis of TEVAR and BMT in acute or subacute uncomplicated TBAD showed a significantly lower incidence of acute stroke in the BMT group, however the risk of late all-cause mortality was significantly higher in the BMT group. The hazard ratios for risk of late all-cause mortality and aorta related mortality were 1.54 and 2.71, respectively, which were significantly higher than those in the TEVAR group [15]. A previous meta-analysis also confirmed the favorable results of uncomplicated and complicated TBAD and showed a significant early mortality advantage of TEVAR over open surgical reconstruction (7.3% versus 19.0%, respectively; *p* = 0.024) [16]. This is due to the lower incidence of paraplegia and stroke with TEVAR [17]. In our study, favorable remodeling and reduction in aortic diameter were observed after TEVAR, which may be related to the fact that 1) the timing of intervention was subacute or early chronic and 2) the aortic diameter was relatively small [4, 18]. In this regard, Nomura et al. provided a cut-off value, recommending intervention at 6.3 months or less after the onset of disease, and at 45 mm or less [19]. Other factors that contribute to good remodeling are still unknown, and further analysis is needed to determine which factors contribute to a good prognosis.

Our study is limited by its retrospective design, relatively small number of patients, incomplete follow-up (6.2%), and varying number of CT scans among patients. Intervention with Preemptive TEVAR is at the discretion of the surgeon and may be subject to selection bias. In some cases, surgical intervention was performed when aneurysmal enlargement was observed at a rate equivalent to 5 mm/6 months before 6 months after onset, so we have included 0.83 mm/month as a criterion for aneurysmal enlargement [7]. These findings highlight the potential of preemptive TEVAR placed in Zone 2 to achieve good outcomes early and prevent future aortic-related events.　This work, however, is hypothesis generating and requires prospective validation in larger cohorts. Although our treatment was performed according to the instructions for use of the stent graft, further studies should be performed to determine which patients have a better prognosis with Zone 2 preemptive TEVAR.

## Conclusions

Zone 2 TEVAR performed in the subacute or early chronic phase for anatomically matched uncomplicated type B aortic dissection had favorable long-term outcomes. Although continuous observation is necessary, preemptive TEVAR seems to be an important option to consider as a treatment strategy in the early stages of dissection, with a lower incidence of long-term aorta-related events compared to conservative treatment.

## Data Availability

The data underlying this article are available in the reasonable request to the corresponding author.

## Abbreviations

TBAD: Stanford type B aortic dissection
TEVAR: Thoracic endovascular aortic repair
BMT: Best medical therapy
ICU: Intensive care unit
HCU: High care unit
CT: Computed tomography
CTA: CT angiography
LSA: Left subclavian artery
IMA: Inferior mesenteric artery
MPR: Multiplanar reconstruction
BMI: Body mass index
COPD: Chronic obstructive pulmonary disease
HTN: Hypertension
NPPV: Noninvasive positive pressure ventilation
RTAD: Retrograde type A dissection
